# Anxiety disorders and eating disorder symptoms: The role of disgust sensitivity

**DOI:** 10.1192/j.eurpsy.2021.1636

**Published:** 2021-08-13

**Authors:** G. Santarelli, M. Innocenti, V. Gironi, V. Faggi, F. Galassi, G. Castellini, V. Ricca

**Affiliations:** 1 Human Health Sciences, University of Florence, Firenze, Italy; 2 Human Health Sciences, University of Florence, firenze, Italy

**Keywords:** eating disorders, disgust, disgust sensitivity, anxiety disorders

## Abstract

**Introduction:**

The comorbidity between anxiety disorders (AD) and eating disorders (ED) has already been well discovered, and because of the link between disgust, food choice and rejection (which appear to be key factors of eating psychopathology), it can be suggested that these three aspects may be related. Accordingly, it can be hypothesized that individuals who express heightened levels of both disgust and eating concerns are at a higher risk to develop an AD. By contrast, disgust, as a negative emotion, influences ED symptoms through a greater level of anxiety.

**Objectives:**

We aimed to investigate the psychopathological role of disgust and eating disorder symptoms in the development and maintenance of anxiety symptoms in patients with AD.

**Methods:**

We enrolled 84 patients admitted in the Psychiatric Unit of Careggi with diagnosis of Anxiety Disorders. We administered them: Zung Anxiety Scale (ZSAS), Disgust Propensity and Sensibility Scale-revised (DPSS-r) and Eating Disorders Examination Questionnaire (EDE-Q).

**Results:**

A multiple regression model having ZSAS total as dependent variable, and EDE-Q total and DPSS disgust sensitivity subscale as independent variables explained 29% of ZSAS total variance (R²=0.290). The effect of EDE-Q total was positive, significant (β=0.331, t=2.631, p=0.011) and explained 11.9% of ZSAS total variance (pr²=0.119). The effect of DPSS disgust sensitivity was positive, significant (β=0.326, t=2.595, p=0.012) and explained 11.6% of ZSAS total variance (pr²=0.116) (Fig.1).

**Figure:**
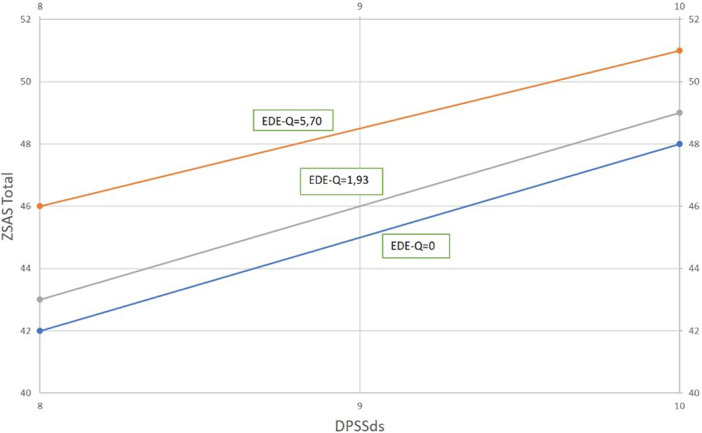

**Conclusions:**

Such preliminary data suggest a possible role of feelings of disgust and eating disorder symptoms in development and maintenance of anxiety symptoms in patients with AD, making them a potential target for psychotherapy.

**Disclosure:**

No significant relationships.

